# Physiological and Ecological Responses of Photosynthetic Processes to Oceanic Properties and Phytoplankton Communities in the Oligotrophic Western Pacific Ocean

**DOI:** 10.3389/fmicb.2020.01774

**Published:** 2020-08-04

**Authors:** Yuqiu Wei, Zhuo Chen, Congcong Guo, Qi Zhong, Chao Wu, Jun Sun

**Affiliations:** ^1^Institute of Marine Science and Technology, Shandong University, Qingdao, China; ^2^Research Centre for Indian Ocean Ecosystem, Tianjin University of Science and Technology, Tianjin, China; ^3^Tianjin Key Laboratory of Marine Resources and Chemistry, Tianjin University of Science and Technology, Tianjin, China

**Keywords:** phytoplankton, photosynthesis, primary production, oceanic properties, Western Pacific Ocean

## Abstract

Understanding the dynamics of primary productivity in a rapidly changing marine environment requires mechanistic insight into the photosynthetic processes (light absorption characteristics and electron transport) in response to the variability of environmental conditions and algal species. Here, we examined the photosynthetic performance and related physiological and ecological responses to oceanic properties [temperature, salinity, light, size-fractionated chlorophyll *a* (Chl *a*) and nutrients] and phytoplankton communities in the oligotrophic Western Pacific Ocean (WPO). Our results revealed high variability in the maximum (F_v_/F_m_; 0.08–0.26) and effective (F_q_′/F_m_′; 0.02–0.22) photochemical efficiency, the efficiency of charge separation (F_q_′/F_v_′; 0.19–1.06), the photosynthetic electron transfer rates (ETR_RCII_; 0.02–5.89 mol e^–^ mol RCII^–1^ s^–1^) and the maximum of primary production [PP_max_; 0.04–8.59 mg C (mg chl *a*)^–1^ h^–1^]. All these photosynthetic characteristics showed a depth-specific dependency based on respective nonlinear regression models. On physiological scales, variability in light absorption parameters F_v_/F_m_ and F_q_′/F_m_′ notably correlated with light availability and size-fractionated Chl *a*, while both ETR_RCII_ and PP_max_ were correlated to temperature, light, and ambient nutrient concentration. Since the presence of nonphotochemical quenching (NPQ_NSV_; 2.33–12.31) and increasing reductant are used for functions other than carbon fixation, we observed nonparallel changes in the ETR_RCII_ and F_v_/F_m_, F_q_′/F_m_′, F_q_′/F_v_′. In addition, we found that the important biotic variables influencing F_v_/F_m_ were diatoms (cells > 2 μm), picosized *Prochlorococcus*, and eukaryotes, but the PP_max_ was closely related to large cyanobacteria (cells > 2 μm), dinoflagellates, and picosized *Synechococcus*. The implication is that, on ecological scales, an interaction among temperature, light, and nutrient availability may be key in driving the dynamics of primary productivity in the WPO, while large cyanobacteria, dinoflagellates, and picosized *Synechococcus* may have a high contribution to the primary production. Overall, the photosynthetic processes are interactively affected by complex abiotic and biotic variables in marine ecosystems, rather than by a single variable.

## Introduction

Current trends of change in oligotrophic marine ecosystems with ongoing climate change include warming, acidification, oligotrophication, and the increases in water column stratification and light penetration (Gao et al., 2019). All of these anticipated changes will inevitably interact to affect the photosynthetic performance of phytoplankton and hence marine primary productivity (Gao et al., 2012; Hoppe et al., 2015; Schuback et al., 2017; Hughes et al., 2018). On physiological scales, these effects can be observed as rapid metabolic adjustments (seconds to hours), while they are manifested as phytoplankton species succession on ecological scales (days to months) (Schuback and Tortell, 2019). To adapt to the changing marine environment, phytoplankton have evolved extreme photophysiological plasticity, ultimately leading to different physiological and ecological responses of photosynthetic processes to environmental variability (Moore et al., 2006; Claquin et al., 2008; Wei et al., 2019b). Accurately evaluating the photosynthetic processes in marine phytoplankton and their capacity to respond to environmental changes is, therefore, relevant to help predict ongoing climate impacts on the dynamics of marine primary productivity.

The photosynthetic processes comprise a series of diverse physiological and biochemical reactions, leading from light absorption via electron transport to carbon fixation (Schuback and Tortell, 2019). In recent years, fast repetition rate fluorometry (FRRF) has been advocated as major means of rapidly estimating the variability of light absorption characteristics and electron transport at unprecedented spatial and temporal resolution (Moore et al., 2003; Smyth et al., 2004; Oxborough et al., 2012; Aardema et al., 2018). Importantly, measurements of these photosynthetic processes can be linked synchronously to measurements of physical and/or chemical variables at the time of sampling (Lawrenz et al., 2013). Although not measuring CO_2_-fixation directly, these FRRF measurements can provide photosynthetic electron transfer rates (ETR_RCII_) of photosystem II (PSII). Thereafter, the ecologically relevant rates of carbon fixation can be converted by FRRF-derived ETR_RCII_ through a conversion factor, i.e., the effective electron requirement for carbon fixation (Melrose et al., 2006; Zhu et al., 2016; Schuback et al., 2017; Morelle and Claquin, 2018). Additionally, the applicability of FRRF-based measurements to estimate marine primary production, alone or in combination with other techniques, are potentially limited since the light absorption characteristics and electron transport vary significantly in response to environmental constraints or combinations thereof and changes in species taxonomy and physiology (Lawrenz et al., 2013; Jin et al., 2016; Schuback et al., 2017; Xie et al., 2018; Wei et al., 2019b; Zhu et al., 2019). As such, more recent studies have sought to better characterize the extent and nature of variation between these photosynthetic processes and environmental/biological variables (Moore et al., 2003; Suggett et al., 2009; Schuback et al., 2017, etc.). If possible, *in situ* measurements of FRRF-derived primary productivity in marine ecosystems can be achieved at the photophysiological level.

Yet to our knowledge, there is no direct experimental investigation in evaluating the variability of photosynthetic processes and in quantifying the primary productivity based on FRRF measurements in the Western Pacific Ocean (WPO) (Richardson et al., 2016). Our goal here is to determine the variability of light absorption characteristics and electron transport [mainly including photosynthetic quantum efficiency (F_v_/F_m_, F_q_′/F_m_′, and F_q_′/F_v_′), functional absorption cross-section (σ_PSII_), nonphotochemical quenching (NPQ), ETR_RCII_], FRRF-derived primary production, and associated oceanic properties [temperature, salinity, light, size-fractionated chlorophyll *a* (Chl *a*), and nutrients] and phytoplankton communities (micro/nano- and picosized classes). With these data, we can test the hypothesis that photosynthetic performance and primary productivity of phytoplankton vary widely across environmental conditions and algal species in the WPO. We can also infer (1) how the photosynthetic processes respond to specific environmental variable and species composition on physiological scales and (2) what is the key in driving the dynamics of WPO primary productivity on ecological scales. Such physiological and ecological insights will be vital roles in improving the parameterization of photosynthetic performance in marine primary production estimates.

## Materials and Methods

### Studied Stations and Sampling

**FIGURE 1:**
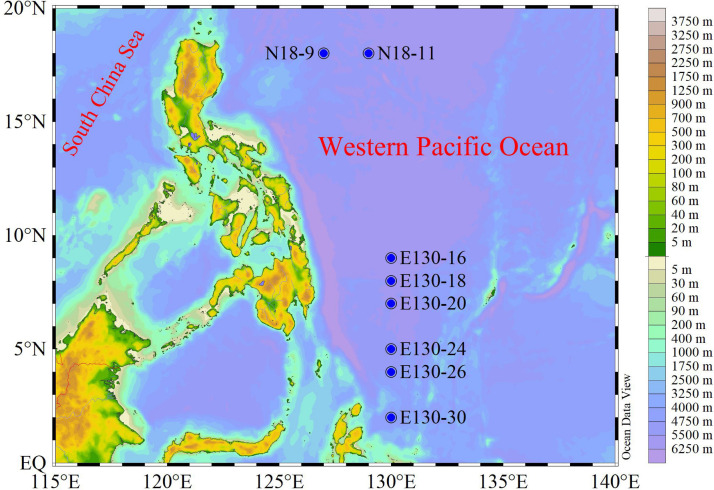
Study area and sampling stations for the Western Pacific Ocean (WPO) cruise during fall. N18-9 and N18-11 denote the northern stations, while E130-16, E130-18, E130-20, E130-24, E130-26, and E130-30 represent the southern stations.

Our experiments were conducted aboard the R/V *Kexue* during a fall cruise (3–28 October 2018) in the WPO (Figure 1). Samples were collected from four to five depths at a total of eight stations; detailed information of stations and sampling are given in Table 1.

**TABLE 1:** Information of stations and sampling depths for biological and environmental parameters during the Western Pacific Ocean (WPO) cruise.

Station	Latitude (°E)	Longitude (°N)	Sampling depths (m)	
			Photosynthetic properties and light irradiance in the upper Z_eu_	Temperature, salinity, nutrients, size-fractionated Chl *a*, and phytoplankton
N18-9	127	18	5, 25, 45, 100	5, 25, 45, 100, 150
N18-11	129	18	5, 25, 45, 100	5, 25, 45, 100, 150
E130-16	130	9	5, 25, 50, 104	5, 25, 50, 104, 150
E130-18	130	8	5, 25, 50, 90	5, 25, 50, 90, 150
E130-20	130	7	5, 25, 50, 100	5, 25, 50, 85, 150
E130-24	130	5	5, 25, 60, 100	5, 25, 60, 100, 150
E130-26	130	4	5, 25, 50, 75	5, 25, 50, 75, 150
E130-30	130	2	5, 25, 50, 78	5, 25, 50, 78, 150

Seawater samples were collected using 12-L Niskin bottles on a rosette equipped with a Sea-Bird Conductivity, Temperature and Depth (CTD) sensor (SBE 19 Plus). Water temperature and salinity were recorded with a CTD system *in situ* at the same time. Seawater samples for FRRF measurements (5–7 ml) were acclimated in low light irradiance for 20 min to allow the oxidation of electron transport chain (ETC) and NPQ relaxation, and then analyzed in shipboard laboratory (Smyth et al., 2004; Suggett et al., 2009). *In situ* light intensity was measured in parallel using an underwater photosynthetically active radiation (PAR, 400–700 nm, μmol quanta m^–2^ s^–1^) sensor (RBR, XRX-620). Samples (1,000 ml) for micro-/nanophytoplankton (cell sizes > 2 μm) analysis were fixed on board with 2% buffered formalin and stored in darkness. Seawater samples (∼2 ml) for picophytoplankton analysis (<2 μm) were incubated in the dark without treatment for 10–15 min at room temperature to avoid loss of resolution and changes in cell counting due to fixation (1% final concentration of paraformaldehyde) or freezing, and then quickly stored at −80°C liquid nitrogen (Jiao et al., 2005; Wei et al., 2019a). Seawater samples for size-fractionated Chl *a* (1,000 ml) were filtered serially through 2 μm × 47 mm nylon membrane and 0.2 μm × 47 mm polycarbonate membrane filters under low vacuum pressure (<0.04 MPa), therewith immediately freeze trapped in liquid nitrogen for further analysis. Filtered samples (0.45 μm, cellulose acetate membrane) for nutrient analysis were frozen at −20°C until processing.

Z_eu_, the euphotic zone depth, defined here as depth with 1% of surface PAR.

### Biological Sample Analysis

All FRRF measurements for PSII photosynthetic properties were conducted on an *in situ* FastOcean sensor with Act2 system (Chelsea Technologies Group, Ltd.). We applied a single-turnover (ST) protocol consisting of 100 flashlets (Fet, a single 1 μs excitation pulse from LEDs) with 2.0 μs Fet pitch to obtain saturation and relaxation sequences. Subsequently, we measured these ST-Fet sequences continuously (2.0-μs interval) throughout the light curve and programmed the length of each light step to make all derived parameters to reach steady state. Blue LED (450 nm) can excite Chl *a* pigments, covering the light absorption spectrum of most photosynthetic algae such as diatoms and dinoflagellates, etc. In mixed phytoplankton communities of the WPO, cyanobacteria mainly use various phycobilin pigments in phycobilisomes to absorb light, instead of Chl *a* (McConnell et al., 2002). However, the phycobilin pigments were excited at longer wavelengths ranging from green and orange/red light. We thus provided the excitation power by LEDs (E_LED_) at three wavelengths centered on 450, 530, and 624 nm to cover the broad range of absorption spectrum to improve the light absorption and generate a saturating pulse, i.e., enough light absorbed to close all PSII reaction centers (RCII). The Rσ_PSII_ values (probability of an RCII being closed during the first Fet saturation phase) reported by Act2 system provided a useful indication of E_LED_ optimization. Ideally, the dark-adapted values of Rσ_PSII_ should fall between 0.05 and 0.07 with any of the LED combinations used. During the cruise, the usable range extended to between 0.03 and 0.08, approximately. At steady state, fluorescence-light response curves were retrieved subsequently by exposing each sample sequentially to 8–12 actinic background irradiances spanning from 0 to 1,000 μmol quanta m^–2^ s^–1^. In addition, the retention time of initial light condition was twice as long as the dark adaptation and subsequent light steps.

Micro-/nanophytoplankton samples were concentrated with 100 ml settlement columns for 24–48 h according to the Utermöhl method (Sun and Liu, 2003; Wei et al., 2017). The taxonomy and abundance of micro-/nanophytoplankton were identified and counted, respectively, under an inverted microscope (Motic BA300) at 200 (or 400) × magnification. According to different fluorescence signals and light-scattering characteristics, picophytoplankton including *Synechococcus* (Syn), *Prochlorococcus* (Pro), and picoeukaryotes (PEuks) were classified and quantified by flow cytometry (BD Accuri C6), respectively, following the standard methods detailed in Jiao et al. (2005) and Wei et al. (2019a). Meanwhile, 2-μm fluorescent beads (Polysciences) were added to 1 ml replicated samples just before analysis as the instrument internal standard.

Nutrients containing ammonium, phosphate, nitrate, nitrite, and silicate were measured by Technicon AA3 Auto-Analyzer (Bran+Luebbe). Dissolved inorganic nitrogen (DIN; the sum of the concentrations of ammonium, nitrite, and nitrate) was analyzed using the method of copper–cadmium column reduction. Dissolved inorganic phosphorus (DIP) and silicate (DSi) were measured using molybdenum blue reagents and standard molybdic acids, respectively (Karl and Tien, 1992; Brzezinski and Nelson, 1995). Furthermore, we imposed a minimum nutrient concentration of 0.01 μmol L^–1^ to avoid issues with detection limits. Size-fractionated Chl *a* filters were extracted in 5 ml 90% acetone (4°C for 24 h). After removal of the filters, Chl *a* concentrations were performed on a CE Turner Designs Fluorometer following the acidification method of Welschmeyer (1994).

### FRRF-Derived Photophysiological Parameters

FRRF-derived photophysiological parameters corresponding to each actinic light level were derived by an iterative nonlinear fitting procedure and recorded from the average of all acquisitions. According to the classical biophysical model of Kolber et al. (1998), the minimum and maximum fluorescence (F) yields for dark-regulated state (F_o_ and F_m_) and for light-regulated state (F′ and F_m_′) were measured, respectively. The functional absorption cross-section of PSII (σ_PSII_ in darkness or σ_PSII_′ under ambient light, Å RCII^–1^) can be determined by parameterizing the fluorescence–light response curve of F yields from F_o_ (F′) to F_m_ (F_m_′). In this way, the maximum [F_v_/F_m_ = (F_m_ - F_o_)/F_m_] and effective [F_q_′/F_m_′ = (F_m_′ - F′)/F_m_′] photochemical efficiency of PSII under dark-adapted and light-regulated states were calculated, respectively, as per Oxborough et al. (2000).

The PSII operating efficiency (F_q_′/F_v_′) quantified the fraction of functional RCII and accounted for the extent of photochemical quenching/(energy conversion) by PSII (i.e., the efficiency of charge separation in RCII) (Suggett et al., 2003; Melrose et al., 2006). NPQ at given light level was derived from normalized Stern–Volmer quenching coefficient, defined as NPQ_NSV_ [NPQ_NSV_ = F_o_′/F_v_′, where F_o_′ represented the minimum F yield in the presence of NPQ_NSV_, was estimated as F_o_′ = F_o_/(F_v_/F_m_ + F_o_/F_m_′)] (Müller et al., 2001; Moore et al., 2003; Xie et al., 2018; Wei et al., 2019b).

(1) $${\text{F}}_{\text{q}}^{{}^{\prime }}/{\text{F}}_{\text{v}}^{{}^{\prime }}=\left({\text{F}}_{\text{m}}^{{}^{\prime }}-{\text{F}}^{{}^{\prime }}\right)/\left({\text{F}}_{\text{m}}^{{}^{\prime }}-{\text{F}}_{\text{o}}^{{}^{\prime }}\right)$$

Our FRRF measurement protocol allowed for reliable estimation of σ_PSII_′ in the existence of NPQ_NSV_. The instantaneous RCII normalized ETR_RCII_ (mol e^–^ mol RCII^–1^ s^–1^) for each light level was calculated as the product of PAR (E, μmol quanta m^–2^ s^–1^), σ_PSII_′ at E, F_q_′/F_v_′ and the constant value (6.022 × 10^–3^) for converting μmol quanta to quanta and Å^2^ (10^–20^ m^2^) to m^2^ according to biophysical sigma-based algorithm (Suggett et al., 2003; Schuback et al., 2015; Xie et al., 2018):

(2) $${\text{ETR}}_{\text{RCII}}=\text{E}\times \sigma_{\text{PSII}}^{\prime }\times \frac{{\text{F}}_{\text{q}}^{{}^{\prime }}}{{\text{F}}_{\text{v}}^{{}^{\prime }}}\times 6.022\times 10^{-3}$$

In this study, the ^14^C-measured data were not collected as part of the experiments included here because the abiotic and/or biotic variables would be lost using a region-specific conversion factor, especially to monitor the physiological responses to environmental changes on primary productivity. However, we measured the charge separation rate per unit volume in PSII [*JV*_PSII_, electrons (PSII m^–3^) s^–1^], which generally correlates well with photosynthetic O_2_ evolution (Oxborough et al., 2012) and can roughly provide an estimate of theoretical maximum of primary production {PP_max_ [mg C (mg chl *a*)^–1^ h^–1^]} (Wei et al., 2019b).

(3) $${\text{PPmax}=k\times JV}_{\text{PSII}}=k\times \sigma_{\text{PSII}}\times \left[\text{RCII}\right]\times \left(1-\text{C}\right)\times {\text{E}}_{\text{LED}}$$

(4) $$\left[\text{RCII}\right]=\frac{{\text{K}}_{\text{R}}}{{\text{E}}_{\text{LED}}}\times \frac{{\text{F}}_{\text{O}}}{\sigma_{\text{PSII}}}$$

where [RCII] is the concentration of PSII reaction centers with units of mol RCII m^–3^; (1-C) is the fraction of RCII in the open state, denoted here as q*^*p*^* [q*^*p*^* = (*F*’-*F*_o_’)/(*F*_m_’-*F*_o_’)]; E_LED_ is the intensity of the fluorometer (photons m^–2^ s^–1^); and K_R_ is a specific constant (photons m^–3^ s^–1^). The specific constant *k* includes the following conversions: 3,600 s h^–1^, 0.25 C quanta^–1^, 12 g C mol^–1^, and 200–950 mol Chl *a* mol RCII^–1^ (Smyth et al., 2004; Suggett et al., 2009; Oxborough et al., 2012).

### Statistical Analyses

Average data are given values ± SD (standard deviation). Spearman correlation analyses (*r*) were used to examine the significant relationship among abiotic and/or biotic parameters (SPSS, V 25). Analysis results were subsequently visualized based on “pheatmap” package in R software (V 3.6.1). The nonlinear regression models (NRMs; Origin V 8.5) and *t-*test (Prism) could provide the curve fit of depth-specific photosynthetic parameters (Lawrenz et al., 2013; Richardson et al., 2016). Statistical significance level was set to 0.05. Abundance of phytoplankton communities was log_10_-transformed to improve the normality. Unless otherwise stated, photosynthetic parameters, phytoplankton abundance, and Chl *a* concentration used for presenting the spatial variation are expressed as depth-weighted averages (as calculated by dividing the trapezoidal integration of measured values for each variable by the maximum sampling depth). The depth-weighted average equation was calculated as (Wei et al., 2019b):

(5) $$A=\left[\sum_{n}^{n+1}\frac{\left(A_{i}+A_{i+1}\right)}{2}\times \left(D_{i+1}-D_{i}\right)\right]/\left(D_{\text{MSL}}-D_{\text{S}}\right)$$

where *A*_i_ is the photosynthetic parameter or phytoplankton abundance (cells L^–1^) at sampling layer *i*; *n* is the number of sampling layers, and *D*_i_ is the depth at sampling layer *i* (m); and *D*_MSL_ and *D*_S_ are the depths of maximum sampling layer (m) and the surface sampling depth (5 m), respectively.

## Results

### Temperature, Salinity, Light Intensity, and Nutrients

**FIGURE 2:**
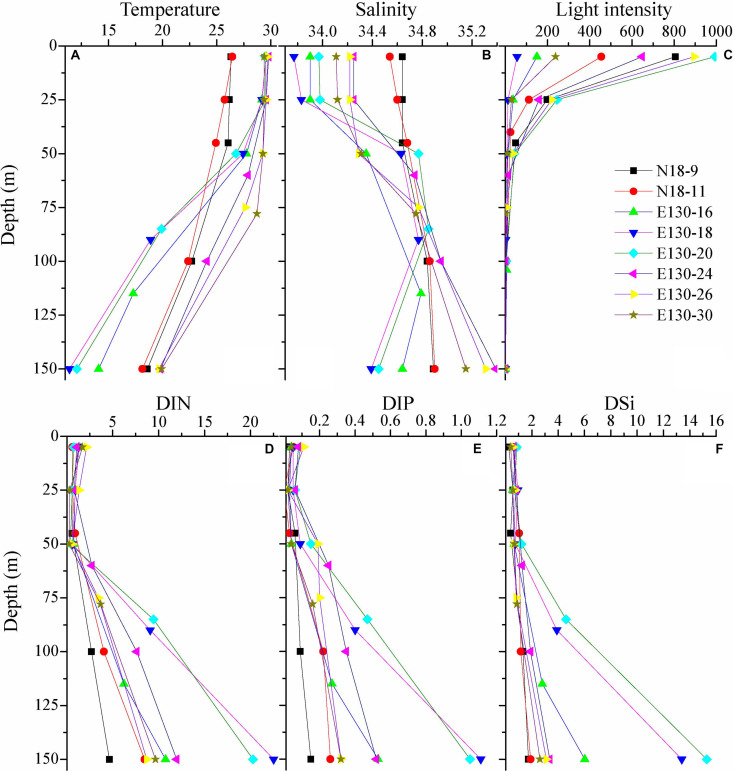
Vertical profiles for **(A)** temperature (°C), **(B)** salinity, **(C)** light intensity (μmol quanta m^– 2^ s^– 1^), and concentrations (μmol L^– 1^) of **(D)** dissolved inorganic nitrogen (DIN), **(E)** dissolved inorganic phosphorus (DIP), and **(F)** dissolved inorganic silicate (DSi). Symbols and colors represent different sampling stations as shown in **(C)**.

Within the upper 50 m, water temperature generally ranged from 26.7 to 29.8°C, except at stations N18-9 and N18-11 where water temperature were relatively lower (approximately 24.9–26.5°C) (Figure 2). However, the salinity observed in the upper 50 m at stations N18-9 and N18-11 (34.5–34.7) were much higher than other sampling stations (<34.4). These results suggest that the contrasting differences of temperature and salinity at stations N18-9 and N18-11 relative to other stations may be potentially affected by the Kuroshio current. Apart from the northern stations N18-9 and N18-11, water temperature rapidly decreased to nearly 11.4–19.9°C from 25 to 150 m across other sampling stations. In particular, the average temperature at stations E130-18 and E130-20 were obviously lower within the upper 150 m (Table 2). Analysis of the satellite altimetry^1^ revealed that a cold eddy was present at stations E130-18 and E130-20. Surface light intensity ranged from 56 to 993 μmol quanta m^–2^ s^–1^ but decreased drastically to 0 μmol quanta m^–2^ s^–1^ at 100–125 m.

**TABLE 2:** Mean values (±SD) of temperature (°C), salinity, and nutrient concentrations (μmol L^–1^) at different sampling stations.

Stations/factors	Temperature	Salinity	DIN	DIP	DSi
N18-9	23.9 ± 3.4	34.7 ± 0.1	1.95 ± 0.73	0.07 ± 0.05	0.85 ± 0.64
N18-11	23.5 ± 3.4	34.7 ± 0.2	2.93 ± 1.42	0.11 ± 0.09	1.09 ± 0.51
E130-16	23.5 ± 7.3	34.3 ± 0.4	3.99 ± 1.44	0.18 ± 0.13	2.16 ± 1.35
E130-18	23.3 ± 7.9	34.3 ± 0.5	6.89 ± 2.42	0.34 ± 0.17	3.92 ± 2.45
E130-20	23.5 ± 7.5	34.4 ± 0.4	6.29 ± 2.71	0.36 ± 0.14	4.53 ± 2.23
E130-24	26.2 ± 4.3	34.7 ± 0.5	4.84 ± 1.82	0.25 ± 0.19	1.59 ± 1.11
E130-26	27.2 ± 4.3	34.6 ± 0.5	3.25 ± 1.23	0.16 ± 0.11	1.18 ± 0.07
E130-30	27.3 ± 4.2	34.5 ± 0.5	3.12 ± 1.91	0.11 ± 0.06	1.02 ± 0.91

*DIN, dissolved inorganic nitrogen; DIP, dissolved inorganic phosphorus; DSi, dissolved inorganic silicate.*

As expected, nutrients were consistently low within the upper 50 m in the WPO (Figures 2D–F). DIN concentration in the upper 50 m ranged from 0.24 to 2.15 μmol L^–1^, with an average of 0.89 ± 0.45 μmol L^–1^. DIP was near the limiting concentration (<0.1 μmol L^–1^) or undetectable within the upper 50 m, averaging 0.06 ± 0.03 μmol L^–1^. DSi was also considerably low, ranging from 0.25 to 1.21 μmol L^–1^ (averaging 0.68 ± 0.21 μmol L^–1^) in the upper 50 m. Due to the influence of cold eddy, average DIN, DIP, and DSi concentrations at stations E130-18 and E130-20 were all relatively higher than other sampling stations (Table 2). In contrast to these eddy-sampled stations, average nutrient concentrations at stations N18-9 and N18-11 were obviously lower as a consequence of the Kuroshio influence.

### Variability in Abundances of Micro-/Nano- and Picophytoplankton and Size-Fractionated Chl *a*

**FIGURE 3:**
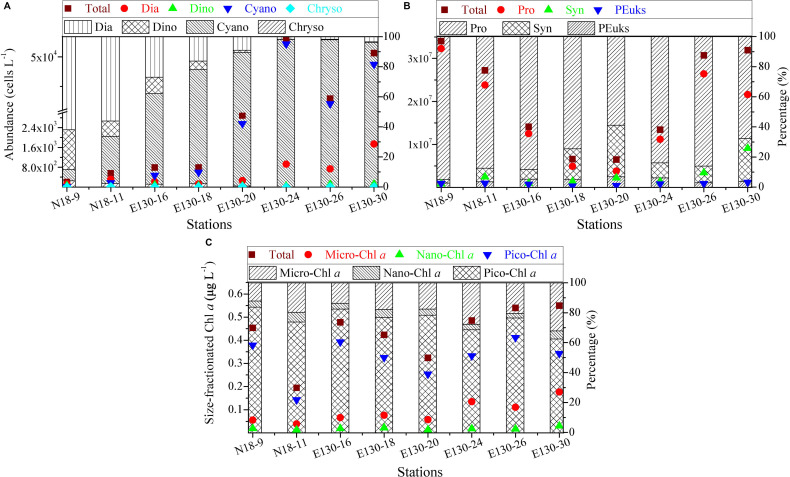
Spatial variations in abundances (cells L^– 1^) of **(A)** micro-/nanophytoplankton and **(B)** picophytoplankton, and in concentrations (μg L^– 1^) of **(C)** size-fractionated Chl *a* along with the relative proportions of their respective components among sampling stations. Symbols and colors represent the depth-weighted averages of phytoplankton abundance and size-fractionated Chl *a* concentration, corresponding to the *y* axis on the left. Note the scale break in **(A)** and logarithmic scale in **(B)**. Different shades denote the fraction of total abundance or total Chl *a* concentration, respectively. Dia, diatoms, Dino, dinoflagellates, Cyano, cyanobacteria, Chryso, chrysophyte; Syn, *Synechococcus*, Pro, *Prochlorococcus*, PEuks, picoeukaryotes; micro-, nano-, and pico-Chl *a* represent micro-, nano-, and picosized phytoplankton Chl *a*, respectively.

Depth-weighted average abundance of the total micro-/nanophytoplankton varied from 0.02 × 10^4^ to 5.32 × 10^4^ cells L^–1^ and averaged at 1.90 ± 0.71 × 10^4^ cells L^–1^ (Figure 3A). The average composition (in terms of abundance) of the micro-/nanophytoplankton community was 70 ± 30% cyanobacteria, 22 ± 12% diatoms, 7 ± 3% dinoflagellates, and 1 ± 1% chrysophyte. Cyanobacteria (mainly containing *R. intracellularis*, *T. thiebautii*, *T. hildebrandtii*, *T. erythraeum*) were the numerically dominant component of the micro-/nanophytoplankton in the WPO. Obvious spatial variations in depth-weighted average abundances of micro-/nano-sized diatoms, dinoflagellates, and cyanobacteria were observed from northern stations to southern stations, with an increase in cyanobacteria, but a decrease in diatoms and dinoflagellates. Species in chrysophyte were recorded more sparsely among all sampling stations, only including *D. fibula*.

Depth-weighted average abundance of the total picophytoplankton was generally between 0.65 × 10^7^ and 3.41 × 10^7^ cells L^–1^ in the WPO, with lower abundance found at stations E130-18 and E130-20 (Figure 3B). Apparently, picophytoplankton abundance was nearly three to five orders of magnitude more abundant than micro/nanophytoplankton, indicating that picophytoplankton contributed a large proportion of the phytoplankton communities. This could be further confirmed by the significant fraction of picosized Chl *a* to the total, averaging 75 ± 7% and ranging from 62 to 84% (Figure 3C). At all stations, Pro (average 1.71 ± 1.04 × 10^7^ cells L^–1^) was typically more abundant than Syn (average 2.69 ± 1.73 × 10^6^ cells L^–1^) and PEuks (average 7.95 ± 2.88 × 10^5^ cells L^–1^). The relative proportions of Pro and Syn to total picophytoplankton abundance averaged 80 ± 12% and 15 ± 9%, respectively, suggesting that the picophytoplankton fraction was primarily characterized by a high abundance of picocyanobacteria (i.e., Pro and Syn).

Depth-weighted average concentration of the total Chl *a* was considerably low in the WPO, averaging 0.43 ± 0.11 μg L^–1^ (range, 0.19–0.55 μg L^–1^, Figure 3C). The Chl *a* concentration in micro-/nanosized fraction (referred to as “micro/nano-Chl *a*”) ranged from 0.05 to 0.21 μg L^–1^ (average, 0.11 ± 0.05 μg L^–1^), and the average contribution of micro-/nano-Chl *a* to the total was 25 ± 7% (range, 16–38%). Picosized Chl *a* (referred to as “pico-Chl *a*”) was typically between 0.14 and 0.41 μg L^–1^, with an average of 0.32 ± 0.09 μg L^–1^. Pico-Chl *a* was two- to fourfold greater than micro-/nano-Chl *a* among stations, thus contributing a significant proportion of the total (∼75%).

### FRRF-Derived Photophysiological Characteristics

**FIGURE 4:**
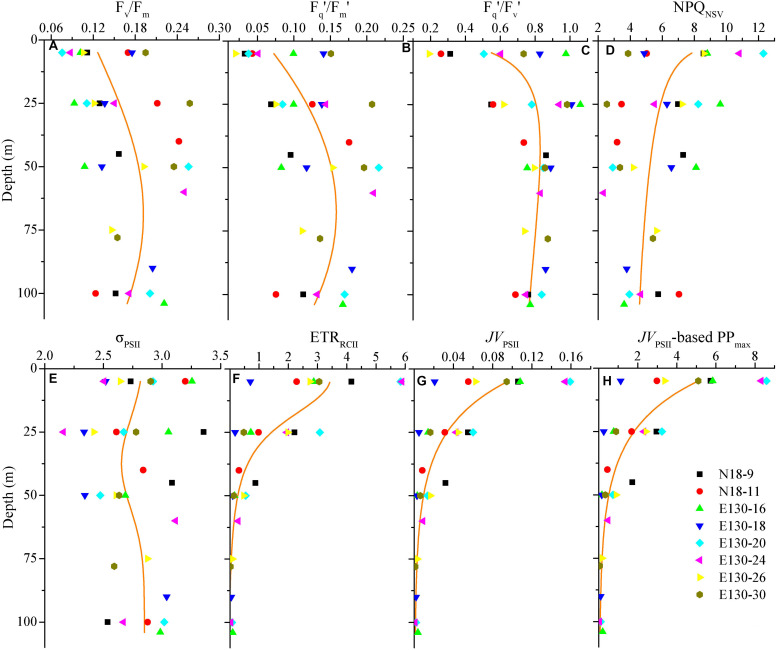
Vertical profiles for photosynthetic parameters of **(A)** F_v_/F_m_, **(B)** F_q_′/F_m_′, **(C)** F_q_′/F_v_′, **(D)** NPQ_NSV_, **(E)** σ_PSII_ (Å RCII^– 1^), **(F)** ETR_RCII_ (mol e^–^ mol RCII^– 1^ s^– 1^), **(G)** *JV*_PSII_ [electrons (PSII m^– 3^) s^– 1^], and **(H)** *JV*_PSII_-based PP_max_ [mg C (mg chl *a*)^– 1^ h^– 1^]. Symbols and colors represent different sampling stations as shown in **(H)**. Solid lines denote the curve fit for the depth dependence of photosynthetic parameters and PP_max_. Note that these curve fit are inferred from all data points of photosynthetic parameters and PP_max_ against depth according to the NRM models shown in Table 3.

**FIGURE 5:**
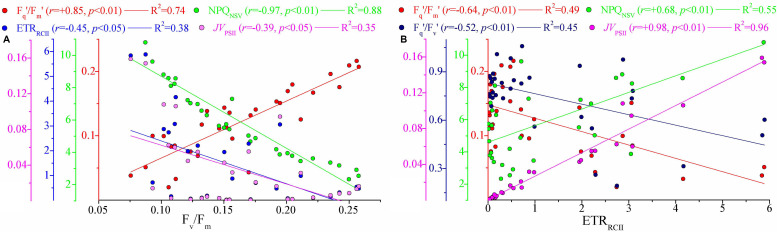
Significant relationships among photophysiological parameters. **(A)** F_v_/F_m_ vs. F_q_′/F_m_′, NPQ_NSV_, ETR_RCII_ (mol e^–^ mol RCII^– 1^ s^– 1^), and *JV*_PSII_ [electrons (PSII m^– 3^) s^– 1^]; **(B)** ETR_RCII_ (mol e^–^ mol RCII^– 1^ s^– 1^) *vs.* F_q_′/F_m_′, NPQ_NSV_, F_q_′/F_v_′, and *JV*_PSII_ [electrons (PSII m^– 3^) s^– 1^]. Lines represent the least square regression, which is statistically significant for both cases (Spearman correlation coefficient *r*-, *p*-value, and regression variance *R*^2^). Symbol color indicates different photosynthetic parameter. The colored *y*-axes correspond to the variability in magnitude of respective photosynthetic parameters. Note difference in *y*-axis scale.

NRM analysis revealed that FRRF-derived photophysiological parameters and primary production (*JV*_PSII_-based PP_max_) varied dramatically with depth in the upper Z_eu_ zone (0.1% surface light level; Figure 4). F_v_/F_m_ was generally between 0.08 and 0.26 (average 0.16 ± 0.05, unitless), with a subsurface maximum of the curve fit found between 50 and 75 m depth (Figure 4A). Overall, F_v_/F_m_ was low throughout the Z_eu_ and among stations. The curve fit for depth dependency of F_q_′/F_m_′ (range, 0.02–0.22 and average 0.12 ± 0.05, unitless) analogously followed the fitting trend observed for F_v_/F_m_ (Figure 4B); this is partly because there was a huge auto-correlation between these two parameters (*r* = 0.85, *p* < 0.01; Figure 5A). F_q_′/F_v_′ showed a different depth-dependence pattern in vertical profile, averaging 0.74 ± 0.21 (range, 0.19–1.06, unitless; Figure 4C). The fitted profile for F_q_′/F_v_′ had yet lower surface values and a shallower subsurface maximum estimating ∼0.81 at 25 m (Model C, Table 3). The variation in F_q_′/F_v_′ across all sampling stations was correlated to the variation in F_q_′/F_m_′(*r* = 0.59, *p* < 0.01; Eq. 1). NPQ_NSV_ was typically between 2.33 and 12.31 and averaged 5.95 ± 2.51 (unitless), and a trend of decreased NPQ_NSV_ with depth was observed in vertical curve fit (Figure 4D). Because of the endogenous changes in metabolic energy allocation, NPQ_NSV_ showed negative correlations with F_v_/F_m_ (*r* = -0.97, *p* < 0.01; Figure 5A) and F_q_′/F_m_′ (*r* = -0.87, *p* < 0.01). Compared to other depth-dependence profiles, σ_PSII_ was less variable (Figure 4E), ranging from 2.16 to 3.36 Å RCII^–1^ with an average value of 2.76 ± 0.29 Å RCII^–1^. There was no meaningful correlation between σ_PSII_ and other photophysiological parameters (*p* > 0.05). At all stations, ETR_RCII_ ranged from 0.02 to 5.89 mol e^–^ mol RCII^–1^ s^–1^ (average, 1.33 ± 1.06 mol e^–^ mol RCII^–1^ s^–1^) within the upper Z_eu_ (Figure 4F). The curve-fitting ETR_RCII_ was generally higher in the surface (∼3.44 mol e^–^ mol RCII^–1^ s^–1^, Model F; Table 3), with a rapid decline at depths deeper than 15–25 m. A positive correlation was observed between ETR_RCII_ and *JV*_PSII_ (*r* = 0.98, *p* < 0.01; Figure 5B), suggesting that the overall *JV*_PSII_ variation was potentially driven by the ETR_RCII_. Similarly, *JV*_PSII_ was maximum at the surface [0.16 electrons (PSII m^–3^) s^–1^] and declined with depth to a minimum value of 0.008 electrons (PSII m^–3^) s^–1^ at 100 m (Figure 4G). The curve fit for depth dependency of *JV*_PSII_ showed a consistent trend with *JV*_PSII_-based PP_max_ (Figure 4H, Eq. 3). Within the upper Z_eu_, *JV*_PSII_-based PP_max_ ranged from 0.04 to 8.59 mg C (mg chl *a*)^–1^ h^–1^, with an average value of 1.92 ± 1.41 mg C (mg chl *a*)^–1^ h^–1^. Both ETR_RCII_ and *JV*_PSII_-based PP_max_ were negatively correlated with the alteration of photochemical efficiency (F_v_/F_m_, F_q_′/F_m_′, F_q_′/F_v_′; *p* < 0.05), but positively correlated with NPQ_NSV_ (*p* < 0.01; Figure 5).

**TABLE 3:** The nonlinear regression model (NRM) models for the curve fit of photosynthetic parameters and PP_max_ vs. depth (*d*) along with the NRM-fitting variance (*R*^2^) and two-tailed *t*-test (*p*).

NRM models	Parametric formulas	*R*^2^	*p*
Model A	ln[(F_v_/F_m_) - 0.01] = -1.7 - (*d* - 69)^2^/9,112	0.31	*p* < 0.0001
Model B	F_q_′/F_m_′ = 256,240/[(*d* - 67)^2^ + 106,227] - 2.25	0.49	*p* < 0.0001
Model C	ln[(F_q_′/F_v_′) - 0.31] = 4.64 - ln(*d*) - [ln(*d*/904.99)]^2^/6.08	0.42	*p* < 0.0001
Model D	NPQ_NSV_ = 10.47*d*^–0.17^	0.34	*p* < 0.0001
Model E	ln(2.85-σ_PSII_) = -1.66 - (*d* - 37)^2^/614.6	0.26	*p* < 0.0001
Model F	ETR_RCII_ = 3.5/(1 + *d*^2.75^/4,935) - 0.0037	0.75	*p* < 0.0001
Model G	*JV*_PSII_ = 42.07/[(*d*+ 3.37)^2^ + 361.5] - 0.0025	0.74	*p* < 0.0001
Model H	*JV*_PSII_-based PP_max_ = 2,270.69/[(*d*+ 3.37)^2^ + 361.5] - 0.132	0.74	*p* < 0.0001

The curve fit in Figures 4A–H are results produced by models A–H, respectively.

**FIGURE 6:**
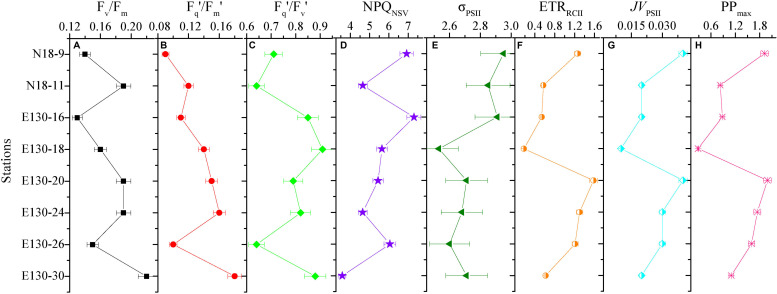
Variability in the depth-weighted averages of fast repetition rate fluorometry (FRRF)-derived photophysiological properties and primary production for each station. **(A)** F_v_/F_m_, **(B)** F_q_′/F_m_′, **(C)** F_q_′/F_v_′, **(D)** NPQ_NSV_, **(E)** σ_PSII_ (Å RCII^– 1^), **(F)** ETR_RCII_ (mol e^–^ mol RCII^– 1^ s^– 1^), **(G)** *JV*_PSII_ [electrons (PSII m^– 3^) s^– 1^], and **(H)** *JV*_PSII_-based PP_max_ [mg C (mg chl *a*)^– 1^ h^– 1^]. The error presented with each depth-weighted average is the 95% confidence interval. Note difference in *x*-axis scale.

Depth-weighted average values of FRRF-derived photophysiological parameters and *JV*_PSII_-based PP_max_ were markedly different across all sampling stations (Figure 6). The depth-weighted average F_v_/F_m_ (unitless) was higher at station E130-30 (0.22), but lower at stations N18-9 and E130-16, 26 (0.11–0.15). However, the spatial variability for depth-weighted average F_v_/F_m_ and F_q_′/F_m_′ were broadly similar (*r* = 0.85, *p* < 0.01). The depth-weighted average NPQ_NSV_ (unitless) was approximately twofold higher at stations N18-9 and E130-16, 26 (6.05–7.31) than at station E130-30 (3.58). At the eddy-sampled station E130-18, F_q_′/F_v_′ was relatively higher, with the depth-weighted average of 0.91 (unitless), whereas σ_PSII_, ETR_RCII_, *JV*_PSII_, and *JV*_PSII_-based PP_max_ were lower than other stations. Among all stations, the spatial variations in depth-weighted averages of ETR_RCII_ and *JV*_PSII_ (*JV*_PSII_-based PP_max_) showed greater similarity (*r* = 0.91, *p* < 0.01): their values were much higher at stations N18-9 and E130-20.

## Discussion

### Physiological and Ecological Responses of Photosynthetic Processes to Oceanic Properties

**FIGURE 7:**
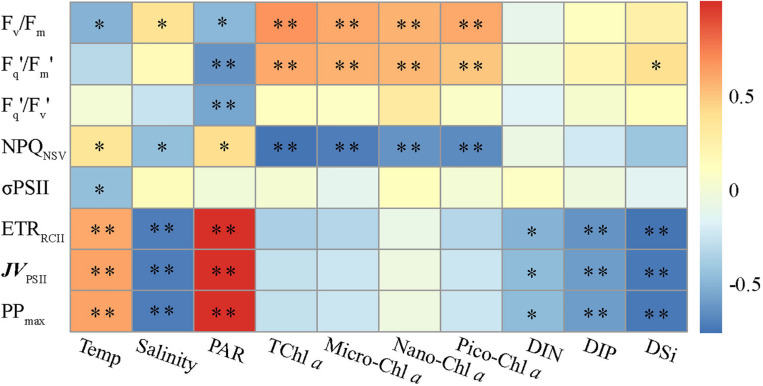
Relationships between photosynthetic parameters and various environmental factors. Spearman correlation coefficients (*r*) ranged from negative to positive and are indicated by color intensity changing from dark blue to red, respectively. ^∗∗^*p* < 0.01; ^∗^*p* < 0.05 (two-tailed). Temp is temperature, TChl *a* is total Chl *a*. Micro-, nano-, and pico-Chl *a* represent micro-, nano-, and picosized phytoplankton Chl *a*, respectively. DIN, dissolved inorganic nitrogen; DIP, dissolved inorganic phosphorus; DSi, dissolved inorganic silicate.

**FIGURE 8:**
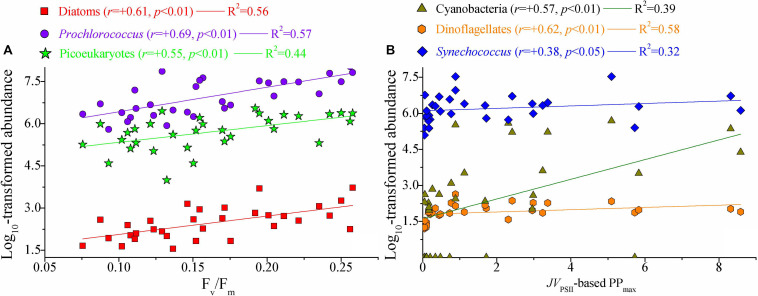
Significant relationships between photosynthetic parameters and natural phytoplankton communities. **(A)** F_v_/F_m_ *vs*. large diatoms, picosized Pro, and PEuks; **(B)** *JV*_PSII_-based PP_max_ [mg C (mg chl *a*)^– 1^ h^– 1^] vs. cyanobacteria, dinoflagellates, and Syn. Lines represent the least square regression, which is statistically significant for both cases (Spearman correlation coefficient *r*-, *p*-value, and regression variance *R*^2^). Symbols and colors represent different phytoplankton populations. Phytoplankton abundance (cells L^– 1^) data were log transformed prior to analysis. Note the difference in *x*-axis scale.

While our dataset is too small to draw general conclusions, our experimental results allow us to gain some physiological and ecological insights into how the dominant environmental constraints and algal species regulate the light absorption characteristics and electron transport and what is the key in driving the dynamics of WPO primary productivity. Light absorption parameters F_v_/F_m_ and F_q_′/F_m_′ showed positive correlations with size-fractionated Chl *a* (*p* < 0.01; Figure 7), suggesting that the light absorption characteristics in photosynthetic process are potentially controlled by the variability in phytoplankton communities (Suggett et al., 2009; Schuback et al., 2017; Zhu et al., 2019; Wei et al., 2019b). Certainly, this result appears to be exemplified to differing degrees by the significant relationships between F_v_/F_m_ and large diatoms (cells > 2 μm), picosized Pro and PEuks (see Figure 8A below). With average DIN/DIP ratio notably less than the 16:1 Redfield ratio (Figure 2 and Table 2), the growth of WPO phytoplankton communities are significantly limited by nutrient availability (Saito et al., 2002). As a physiological consequence of nutrient limitation (Oxborough et al., 2012; Jin et al., 2016; Zhu et al., 2016), F_v_/F_m_ and F_q_′/F_m_′ were considerably low throughout the Z_eu_ and among stations (Figures 4, 6). From a photophysiological point of view, the photochemical efficiency in natural phytoplankton assemblages is indirectly affected by the nutrient level (Moore et al., 2006; Rabouille and Claquin, 2016; Schuback et al., 2016). Therefore, the variation in magnitude of F_v_/F_m_ and F_q_′/F_m_′ can be used as a predictor for nutrient use efficiency of marine ecosystems across considerable environmental gradients. However, only nutrient availability is inadequate to explain and predict the magnitude and variability of these derived light absorption parameters (Claquin et al., 2008; Lawrenz et al., 2013). Typically, variability in irradiance level was another primary driver of variability in F_v_/F_m_, F_q_′/F_m_′, and F_q_′/F_v_′ in the WPO (*p* < 0.05; Figure 7). The depth-specific fitting values of these light absorption characteristics we observed were higher at the subsurface (Figure 4), and one important explanation for this is that the interactive effects of light and nutrient levels lead to an increase in these light absorption parameters (Moore et al., 2006; Suggett et al., 2009; Zhu et al., 2019). In contrast, the strong effects of excess irradiance pressure and limitation by nutrients in the surface inhibited the F_v_/F_m_, F_q_′/F_m_′, and F_q_′/F_v_′ (Figure 4; Schuback et al., 2017; Wei et al., 2019b). Consistent with previous observations (Melrose et al., 2006; Claquin et al., 2008; Jin et al., 2016; Xie et al., 2018, etc.), variability in temperature exerted an evident influence on F_v_/F_m_ and σ_PSII_ (*p* < 0.05; Figure 7). The fact that F_v_/F_m_ and σ_PSII_ varied as a function of temperature does not necessarily imply a direct temperature effect on F_v_/F_m_ and σ_PSII_, as temperature can affect other photosynthetic complexes (Richardson et al., 2016). For instance, moderate heat stress is critical for the activity of RuBisCo enzyme in photosynthetic process (Jensen, 2000). Overall, on ecological scales, water temperature, light, and nutrient availability are important environmental variables in regulating the light absorption process.

NPQ_NSV_ was negatively correlated with the light absorption parameters F_v_/F_m_ and F_q_′/F_m_′ (*p* < 0.01; Figure 5), indicating that the photochemical efficiency in electron transport process may be limited by the expression of NPQ_NSV_ (alleviating excess energy pressure and minimizing the potential for photooxidative damage) (Müller et al., 2001; Schuback et al., 2016; Zhu et al., 2016). Indeed, we observed that NPQ_NSV_ was significantly correlated with PAR (*p* < 0.05; Figure 7), further demonstrating the strong effect of irradiance levels on the photosynthetic processes. As expected, we simulated a higher NPQ_NSV_ for surface phytoplankton assemblages (Figure 4), reflecting photophysiological adaptation to optimize photosynthesis under high irradiance level (Müller et al., 2001; Gao et al., 2012; Aardema et al., 2018). Nonparallel changes in the ETR_RCII_ and F_v_/F_m_, F_q_′/F_m_′, and F_q_′/F_v_′ (*p* < 0.05; Figure 5) imply a decoupling of light absorption at the level of RCII and electron transport in ETC, since the presence of NPQ_NSV_ and increasing reductant are used for functions other than carbon fixation (Behrenfeld et al., 2002; Richardson et al., 2016). Due to excess irradiance energy in the surface water, the processes regulating electron transport and preventing overreduction in ETC are closely associated with the expression of NPQ_NSV_ (Smyth et al., 2004; Hughes et al., 2018). It is apparent that the NPQ_NSV_ process can effectively achieve energy-allocation balance, providing mechanistic insight into the decoupling of photosynthetic electron transport and carbon fixation and even the NPQ_NSV_-based primary production (Schuback et al., 2015, 2016; Wei et al., 2019b). Thus, ETR_RCII_ and *JV*_PSII_-based PP_max_ were closely correlated to NPQ_NSV_ (*p* < 0.01; Figure 5). In other words, changes in ETR_RCII_ and *JV*_PSII_-based PP_max_ can be attributed to the NPQ_NSV_ process. Both ETR_RCII_ and *JV*_PSII_-based PP_max_ showed significant temperature and light-dependent responses in natural phytoplankton assemblages (*p* < 0.01; Figure 7), suggesting that temperature and light are determinants in regulating the dynamics of ETR_RCII_ and PP_max_. Based on this, we can thus conclude that the fitting trends of decreased ETR_RCII_ and PP_max_ with depth were controlled by temperature and light (Figure 4). Phytoplankton are acclimated to the high and variable light conditions of the surface layer to alleviate excess energy pressure through faster reoxidation of Q_A_^–^ and a larger PQ pool (Schuback et al., 2017), resulting in the higher ETR_RCII_ we observed. On the other hand, photo-acclimation to lower irradiance stimulates an increase in Chl *a* per cell near the subsurface, which, in turn, decreases the Chl *a*-normalized PP_max_ (Behrenfeld et al., 2002; Moore et al., 2006). Nutrients also had a potential effect on the variations in ETR_RCII_ and PP_max_ (*p* < 0.05; Figure 7), but negatively. This result is in good agreement with previous findings of Richardson et al. (2016) who found both the maximum rate of photosynthesis and the slope of the photosynthesis *vs.* light curve are negatively correlated with ambient nutrient concentration, thus indicating a possible influence of an interaction among light, temperature, and nutrient availability on ETR_RCII_ and PP_max_. Collectively, these tight endogenous and exogenous regulations of the photosynthetic processes upstream from carbon fixation allow phytoplankton assemblages to balance light absorption with electron flow, electron transport, and carbon fixation (Müller et al., 2001; Murata et al., 2007; Schuback et al., 2017).

### Photosynthetic Processes in Relation to Natural Phytoplankton Communities

Marine primary production estimates are highly dependent on assumptions regarding the photosynthetic potential of the resident phytoplankton communities (Richardson et al., 2016). Little is known, however, about the physiological and ecological responses of photosynthetic processes to natural phytoplankton populations. Such physiological and ecological effects of the photosynthetic response in relation to natural phytoplankton communities are clearly evident in our dataset (Figure 8). Light absorption parameter F_v_/F_m_ was clearly correlated with large diatoms (cells > 2 μm), Pro and PEuks (*p* < 0.01). In marine ecosystems, F_v_/F_m_ is known to vary systematically among taxonomic groups, but the highest recorded F_v_/F_m_ values (∼0.65–0.70) are measured for large diatoms (Suggett et al., 2003). The light harvesting antennas of diatoms are known as Chl *a*/*c* and fucoxanthin (Fx) binding proteins, or FCPs, and enable diatoms to efficiently adapt to rapidly changing light intensity. Recently, Pi et al. (2019) reported the structure of PSII-Fx Chl *a*/*c* binding protein supercomplex (PSII-FCPII) from the diatom *Chaetoceros gracilis*, and revealed that the distinct pigment-protein network of the PSII-FCPII supercomplex contributes to efficient light energy harvesting in the diatoms. In the present study, therefore, variation in F_v_/F_m_ was closely associated with the large diatoms. There are two NPQ mechanisms in the diatoms, one associated with antenna units attached to PSII and the other associated with antenna units that detach from PSII (Miloslavina et al., 2009). In particular, the bindings of Chl *c* and Fx further enhance the capabilities of these NPQ mechanisms to dissipate excess energy when necessary (Wang et al., 2019), potentially resulting in the decoupling between large diatoms and *JV*_PSII_-based PP_max_ on physiological and ecological scales. However, none of these NPQ mechanisms would be expected to affect the maximum photochemical efficiency (F_v_/F_m_) of photosynthesis in diatoms (Torres et al., 2014). Picocyanobacteria Pro is also characterized by relatively high values of F_v_/F_m_ (0.55–0.65) (Bruyant et al., 2005). Values of F_v_/F_m_ for picosized PEuks (e.g., *Aureococcus anophagefferens*) are typically between 0.3 and 0.4 (Suggett et al., 2009). Although F_v_/F_m_ values in excess of 0.60 to 0.65 have been measured for some specific species of large cyanobacteria (i.e., *Cyanotheca* and *Anabaena*), values can be as low as 0.1–0.4 for most micro-/nanosized cyanobacteria (Berman-Frank et al., 2003). Phycobiliprotein (PBP) plays an exceptional role in light harvesting in cyanobacteria, but PBPs harvest light in the region of 490–650 nm where the Chl and carotenoids have poor light absorption properties (Campbell et al., 1998). On the other hand, the relatively low F_v_/F_m_ values in large cyanobacteria may be attributed to the substantial phycocyanin concentrations, from which the fluorescence emission band overlaps with that from Chl *a* and, hence, leading to lower values for F_v_/F_m_ (McConnell et al., 2002). However, picocyanobacteria Syn with low concentrations of phycocyanin still has relatively low values of F_v_/F_m_ (Suggett et al., 2009). Overall, these significant correlations between F_v_/F_m_ and algal species may be driven by photoacclimation or a number of evolutionary selection pressure related to the light absorption and energy transfer. The functional and structural advantages in the photosystems of the dominant algal species provide another possible rationale for the intimate correlations between F_v_/F_m_ and algal species (Moore et al., 2003, 2006). Thus, it is not surprising that F_v_/F_m_ correlated with large diatoms and Pro given they were the dominant components of micro-/nano- and picosized phytoplankton communities, respectively (Figure 3). As with F_v_/F_m_, F_q_′/F_m_′ showed significant relations with large diatoms, picosized Pro, and PEuks (*p* < 0.01). The implication is that large diatoms, picosized Pro, and PEuks are the keys in driving the light absorption process.

*JV*_PSII_-based PP_max_ was markedly associated with micro-/nanosized cyanobacteria and dinoflagellates, and picocyanobacteria Syn (*p* < 0.05). We suggest that these algal species may contribute significantly to the WPO primary production on ecological scales. Recently, the orange carotenoid protein (OCP), a carotenoid binding protein, has been found to exist quite widely in marine cyanobacteria, which has an advantage in coping with the excess excitation energy over other algae (Bailey and Grossman, 2008; Sedoud et al., 2014). We thus speculate that the presence of OCP may effectively regulate energy dissipation downstream of light absorption and improve the photosynthetic efficiency through more robust excitation energy transfer. In addition, NPQ_NSV_ in cyanobacteria is triggered by strong blue light, with almost no induction at wavelengths above 520 nm, the utilization of harvested light energy in cyanobacteria becomes more efficient (Bailey and Grossman, 2008). The significant relationship we found between *JV*_PSII_-based PP_max_ and dinoflagellates and Syn agrees well with previous findings of Richardson et al. (2016) who found the dinoflagellates and Syn associated with a higher PP_max_ (the maximum rate of photosynthesis) than large diatoms. Differently, our earlier work in the Bay of Bengal has suggested that the variability in large diatoms and cyanobacteria appeared to be the major drivers of variability in gross primary production (Wei et al., 2019b). Given the wide diversity of the phytoplankton communities in marine ecosystems, therefore, we cannot use the region-specific relationships we find here as being universal for the global ocean. Richardson et al. (2016) proposed an explanation for the relatively large contribution of dinoflagellates to PP_max_ that a small number of dinoflagellates with large biovolume can provide the dominant biovolume in the phytoplankton communities, resulting in a greater increase in light absorption capability and energy transfer efficiency. This involves changes in many relevant biochemical or physiological processes such as package effect, area of photosynthetic membrane space available, cellular resources required for the production of RCII, and membrane intrinsic antennae (Suggett et al., 2009). Another possibility, to some extent, is that some dinoflagellates under natural environments may have more efficient photosynthesis than other algal species. As discussed above, large diatoms and picocyanobacteria Pro showed a relatively high F_v_/F_m_ but were not associated with PP_max_. This fits well with the fact that F_v_/F_m_ was negatively correlated with *JV*_PSII_-based PP_max_ (*r* = -0.39, *p* < 0.05), since the presence of NPQ_NSV_ process and additional reductant are used for functions. Additionally, this negative correlation may arguably be the results of photoacclimation and the influences of temperature and nutrient concentration. That photosynthetic parameters are closely related to different taxonomic groups provides some implications for improving the parameterization of the factors influencing photosynthetic potential, although not universal for the global ocean under all conditions. It is well known that the physical processes in the ocean play important roles in affecting primary production (Falkowski et al., 1991; Furuya et al., 1998). In the present study, however, we did not find any evidence that the Kuroshio and cold eddy have specific or one-way effects on the photosynthetic performance in the WPO. This is arguably because the photosynthetic processes are interactively influenced by complex abiotic and biotic variables in marine ecosystems, rather than by a single variable.

## Data Availability Statement

All datasets presented in this study are included in the article/supplementary material.

## Author Contributions

JS and YW designed the experiment. YW and QZ collected the samples. YW, ZC, CG, and QZ performed the sample analysis. YW wrote the manuscript, with contribution from all authors. All authors read and approved the final manuscript.

## Conflict of Interest

The authors declare that the research was conducted in the absence of any commercial or financial relationships that could be construed as a potential conflict of interest.

## Footnotes

1 http://icdc.cen.uni-hamburg.de/1/daten/ocean/ssh-aviso/
